# Screening of possible biomarkers and therapeutic targets in kidney renal clear cell carcinoma: Evidence from bioinformatic analysis

**DOI:** 10.3389/fonc.2022.963483

**Published:** 2022-10-13

**Authors:** Longfei Peng, Zhangjun Cao, Qi Wang, Lu Fang, Songbai Yan, Dian Xia, Jinyou Wang, Liangkuan Bi

**Affiliations:** Department of Urology, Second Hospital of Anhui Medical University, Hefei, China

**Keywords:** microarray datasets, differentially expressed genes, Protein-protein interaction network, hub gene, renal cell carcinoma (RCC) clear cell renal cell carcinoma (CCRCC)

## Abstract

Renal cell carcinoma (RCC), as one of the most common urological malignancies, has many histologic and molecular subtypes, among which clear cell renal cell carcinoma (ccRCC) is one of the most common causes of tumor-related deaths. However, the molecular mechanism of ccRCC remains unclear. In order to identify the candidate genes that may exist in the occurrence and development of ccRCC, microarray datasets GSE6344, GSE16441, GSE36895, GSE53757 and GSE76351 had been downloaded from Gene Expression Omnibus (GEO) database. Apart from that, the differentially expressed genes (DEGs) were screened through Bioinformatics & Evolutionary Genomics. In addition, the protein-protein interaction network (PPI) was constructed, and the module analysis was performed using STRING and Cytoscape. By virtue of DAVID online database, GO/KEGG enrichment analysis of DEGs was performed. Consequently, a total of 118 DEGs were screened, including 24 up-regulated genes and 94 down-regulated genes. The plug-in MCODE of Cytoscape was adopted to analyze the most significant modules of DEGs. What’s more, the genes with degree greater than 10 in DEGs were selected as the hub genes. The overall survival (OS) and disease progression free survival (DFS) of 9 hub genes were analyzed through GEPIA2 online platform. As shown by the survival analysis, SLC34A1, SLC12A3, SLC12A1, PLG, and ENO2 were closely related to the OS of ccRCC, whereas SLC34A1 and LOX were closely related to DFS. Among 11 SLC members, 6 SLC members were highly expressed in non-cancerous tissues (SLC5A2, SLC12A1, SLC12A3, SLC34A1, SLC34A2, SLC34A3). Besides, SLC12A5 and SLC12A7 were highly expressed in ccRCC. Furthermore, SLC12A1-A7, SLC34A1 and SLC34A3 were closely related to OS, whereas SLC12A2/A4/A6/A7 and SLC34A1/A3 were closely related to DFS. In addition, 5 algorithms were used to analyze hub genes, the overlapping genes were AQP2 and KCNJ1. To sum up, hub gene can help us understand the molecular mechanism of the occurrence and development of ccRCC, thereby providing a theoretical basis for the diagnosis and targeted therapy of ccRCC.

## Introduction

RCC accounts for 4% of adult malignancies. According to the statistics, the probability of males suffering from RCC is 2.2%, and the probability of females suffering from RCC is 1.2% (1). Most of RCC is originated from renal tubular epithelial cells, accounting for more than 90%. ccRCC, as the most common type of RCC, accounts for 40% to 80% of the total RCC. Although some progress has been made in recent years, metastatic ccRCC is usually an incurable malignancy with five-year survival rate of < 20% (2). In fact, ccRCC can be cured by the early diagnosis, but when the disease progresses and metastases, it will belong to the urinary system tumor with the worst prognosis. At present, antiangiogenic therapy and immune checkpoint inhibition therapy are regarded as the very promising comprehensive therapies for the treatment of advanced and metastatic diseases. At the same time, many exploratory studies have identified the epigenetic markers based on DNA methylation, histone modification and ncRNA expression, which epigenetically regulate gene expression in ccRCC (3). Although study related to the molecular mechanism of tumorigenesis and development of ccRCC is conducive to determining new therapeutic targets, its molecular mechanism remains unclear (4). Therefore, it is of great significance to understand the accurate molecular mechanism of occurrence, proliferation, recurrence and metastasis of ccRCC, so as to determine more effective and accurate diagnosis and treatment strategies.

In recent years, with the development of microarray technology and bioinformatic analysis, it has been widely applied to screen DEGs and identify the molecular mechanism of tumorigenesis and development. In this study, five microarray databases were downloaded from GEO, and the DEGs between cancerous and non-cancerous tissues were analyzed in ccRCC. Subsequently, its molecular function and pathway mechanism were understood through GO, KEGG enrichment analysis and PPI network diagram. As a result, a total of 118 DEGs and 15 hub genes were found, which may become the candidate genes for targeted therapy in ccRCC.

## Materials and methods

### Microarray data

Five gene expression databases were downloaded from the GEO database (www.ncbi.nlm.nih.gov/gds/). To be specific, GSE6344 dataset (21212 gene) contained 10 non-cancer samples and 10 ccRCC tissue samples (5). GSE16441(27990 gene) dataset included 17 non-cancer samples and 17 ccRCC tissue samples (6). GSE36895 (46692 genes) dataset contained 25 non-cancer samples and 21 ccRCC tissue samples (7). GSE53757 dataset (46658 genes) contained 72 non-cancer samples and 72 ccRCC tissue samples (8). GSE76351 dataset (25172 genes) contained 12 non-cancer and 12 ccRCC tissue samples.

### Screening of DEGs

The GEO accession was searched through GEO2R (www.ncbi.nlm.nih.gov/geo/geo2r/), and cancer tissues were compared with non-cancer tissues. On this basis, relevant data information was extracted. Beyond that, inclusion criteria were set: 1) logFC (fold Change) ≥2 or logFC (fold change) ≤ -2. 2) adj. P-value <0.01. The genes, which met the standard of each gene expression datasets, were screened. Moreover, venn map was used to obtain DEGs.

### GO and KEGG enrichment analyses of DEGs

Enrichment analysis was carried out using DAVID online database (https://david.ncifcrf.gov/summary.jsp). GO enrichment analysis could be divided into three categories (BP, biological process; CC, cellular component; MF, molecular function). What’s more, the P value of the top 10 hub genes was selected, and the bar chart was used for visual analysis.

### PPI network

Interaction between genes and PPI networks was predicted using the STRING online database (https://cn.string-db.org/cgi/). By analyzing the interaction between proteins, the molecular mechanism of disease could be understood in depth. Cytoscape (v.3.8.2) was used to further process the network diagram processed by STRING online database. Meanwhile, the plug-in MCODE of Cytoscape was used to further analyze the network of DEGs, so as to obtain the most significant module. Inclusion criteria: degree cut-off=2, node score cut-off=0.2, Max depth=100 and k-score=2.

### Hub genes analysis

Genes with degree greater than 10 were considered the hub genes. Apart from that, the co-expression analysis of hub genes was performed using GeneMANIA online platform (http://genemania.org). Statistical analysis and visualization of hub genes were performed by R (v.3.6.3). The UCSC online database was used to construct the hierarchical clustering of hub genes (http://genome-cancer.ucsc.edu). Then, the overall survival of hub genes was performed using Kaplan-Meier curve in GEPIA2 online platform (http://gepia2.cancer-pku.cn/). In cytohubba plug-in, the top 10 hub genes were obtained through five kinds of algorithms of overlapping genes based on venn map analysis. Besides, GEPIA2 was also to analyze the expression of hub genes.

## Results

### Identification of DEGs in ccRCC

**Figure 1:**
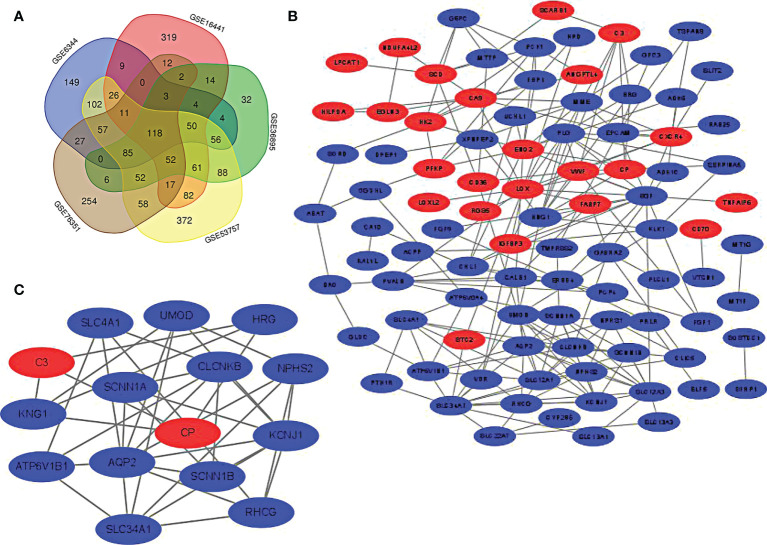
**(A)** screening of DEGs. The genes meeting the standard of each microarray datasets were screened. Veen diagram was made. Overlapping genes were recognized as DEGs. **(B)** PPI network of DEGs was constructed using Cytoscape. **(C)** The most statistically different module was built in PPI network through MCODE plug-in(15 nodes, 37 edges). The up-regulated gene was marked red and the down-regulated gene was marked green.

Five microarray datasets were screened according to the inclusion criteria, and the inclusion expression gene was identified. The overlap of the 5 datasets was considered DEGs and presented in Venn Map (**Figure 1A**). The DEGs included 24 up-regulated genes and 94 down-regulated genes.

### PPI network construction and module analysis

The PPI network graph of DEGs was created through STRING and Cytoscape. As displayed in figure, the up-regulated genes are shown in red, while the down-regulated genes are shown in blue (**Figure 1B**). The most significant module (15 nodes, 37 edges) in DEGs is formed using Cytoscape’s plug-in MCODE (**Figure 1C**).

### GO and KEGG enrichment analyses of DEGs

**Figure 2:**
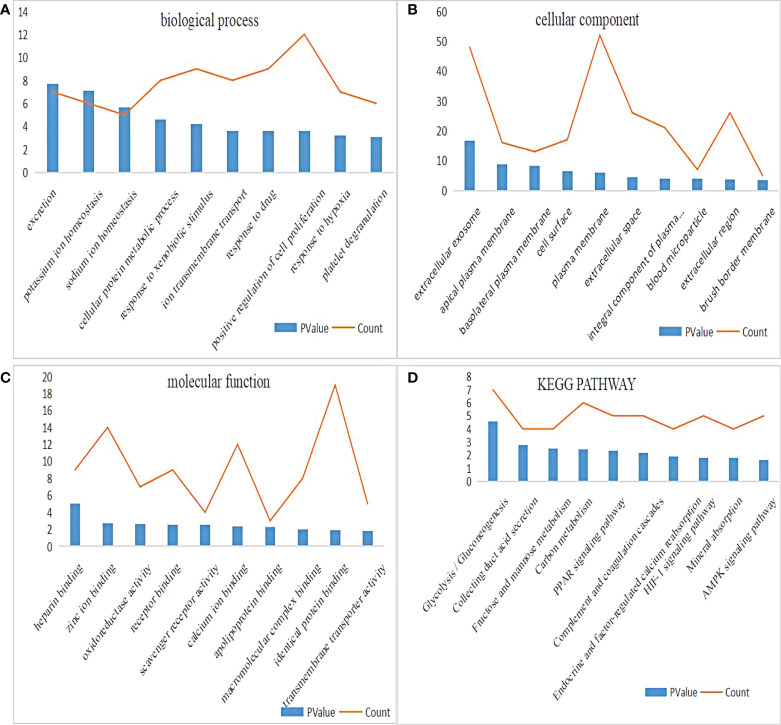
GO and KEGG pathway enrichment analysis of DEGs. **(A)** The biological process enrichment results of DEGs ranked top 10 in P value. **(B)** The cellular component enrichment results of DEGs ranked top 10 in P value. **(C)** The molecular function enrichment results of DEGs ranked top 10 in P value. **(D)** The KEGG pathway enrichment results of DEGs ranked top 10 in P value.

DAVID was used for enrichment analysis, in which the biological classification, function and pathway of DEGs were analyzed. According to the GO analysis, the top 5 changes in biological processes in DEGs were excretion, potassium ion homeostasis, sodium ion homeostasis, cellular protein metabolic process, and response to xenobiotic stimulus (**Figure 2A**). Meanwhile, the top 5 changes in cellular component in DEGs were extracellular exosome, apical plasma membrane, basolateral plasma membrane, cell surface and plasma membrane (**Figure 2B**). In addition, the top 5 changes in molecular function in DEGs were heparin binding, zinc ion binding, oxidoreductase activity, receptor binding and scavenger receptor activity (**Figure 2C**). As shown by KEGG pathway analysis, the top 5 changes were concentrated in glycolysis/gluconeogenesis, thus collecting duct acid secretion, fructose and mannose metabolism, carbon metabolism and PPAR signaling pathway (**Figure 2D**).

### Hub gene screening and analysis

**Figure 3:**
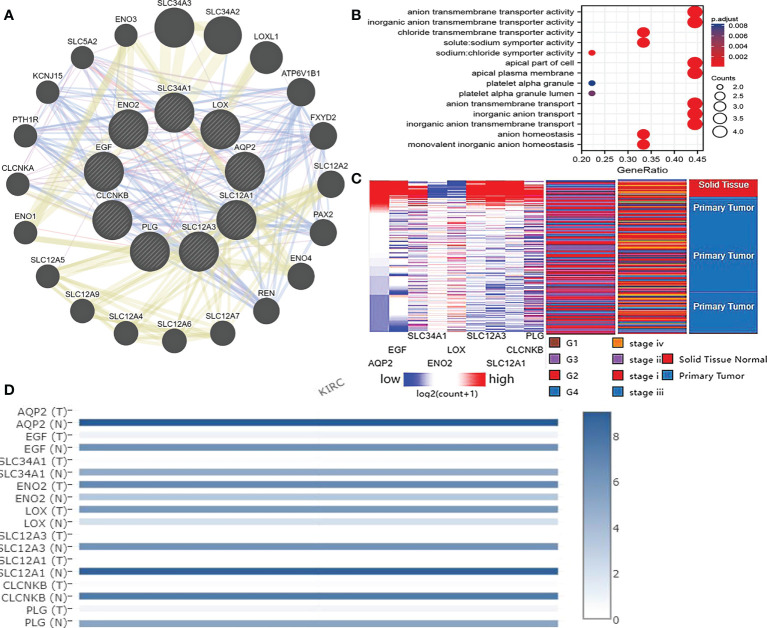
**(A)** coexpression analysis of hub genes. Blue line: Co-localization, Purple line: Co-expression, Yellow line: Shared protein domains, Red line: Physical Interactions. **(B)** Hub gene enrichment analysis results. **(C)** Hierarchical clustering of hub genes was constructed using UCSC. Red represents up-regulation, while blue represents down-regulation. **(D)** Expression of hub genes in ccRCC tissue samples and non cancer tissue samples.

**Figure 4:**
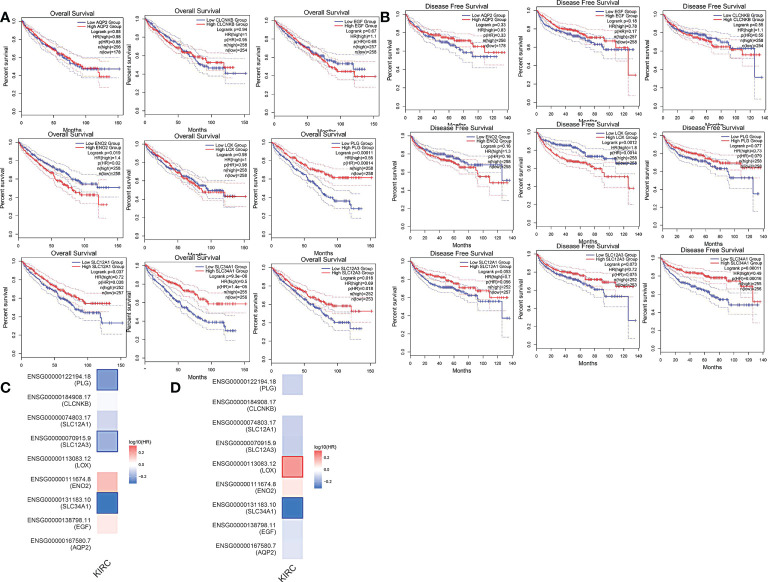
**(A)** overall survival curve of hub genes. **(B)** Disease-free survival curve of hub genes. **(C, D)** OS and DFS heat map analysis of hub genes.

More than 10 degrees in DEGs were screened as hub genes. A total of 9 hub genes had been screened, among which 2 genes were up-regulated in ccRCC tissues (ENO2,LOX), whereas the remaining 7 genes (AQP2, EGF, SLC34A1, SLC12A3, SLC12A1, CLCNKB, PLG) were overexpressed in non-cancerous tissues (**Table 1**). The co-expression network of hub genes was analyzed using GeneMANIA online platform (**Figure 3A**). Enrichment analysis of the biological process of hub gene is presented in **Figure 3B**. Function is mainly concentrated in anion transmembrane transport, inorganic anion transmembrane transporter activity and chloride transmembrane transporter, etc. What’s more, hierarchical cluster analysis revealed the relationship between hub genes expression and clinical stage and grade of ccRCC (**Figure 3C**). The expression of hub genes in non-cancerous tissues and ccRCC tissues is shown in **Figure 3D**. Moreover, the OS and DFS of hub genes were analyzed by GEPIA2 online platform. According to the survival analysis, SLC34A1, SLC12A3, SLC12A1, PLG, and ENO2 were closely related to the OS of ccRCC (**Figure 4A**), whereas SLC34A1 and LOX were closely related to DFS (**Figure 4A**). The analysis results of the survival heat map are shown in **Figures 4C, D**.

**Table 1:** Functional roles of 9 hub genes.

Gene	Full name	Function
AQP2	aquaporin 2	encodes a water channel protein.
EGF	epidermal growth factor	Genetic disorders are associated with the growth and development of certain cancers.
SLC34A1	solute carrier family 34	Gene mutations associated with hypophosphatemia, kidney stones/osteoporosis 1.
ENO2	enolase 2 (gamma, neuronal)	Alpha enolase is converted to Gamma enolase in the neural tissue of mice and primates.
LOX	lysyl oxidase	The protein encoded by this gene is an extracellular copper enzyme that initiates the crosslinking of collagens and elastin.
SLC12A3	solute carrier family 12 , member 3	This gene encodes a renal thiazide-sensitive sodium-chloride cotransporter that is important for electrolyte homeostasis.
SLC12A1	solute carrier family 12 , member 1	Urine concentration plays a key role in the absorption of sodium chloride is the main reason.
CLCNKB	Chloride channel, voltage-sensitive Kb	Regulation of cell volume, membrane potential stability, signal transduction and transmembrane transport.
PLG	plasminogen	The protein is a secreted blood zymogen that is activated by proteolysis and converted to plasmin and angiostatin.

**Figure 5:**
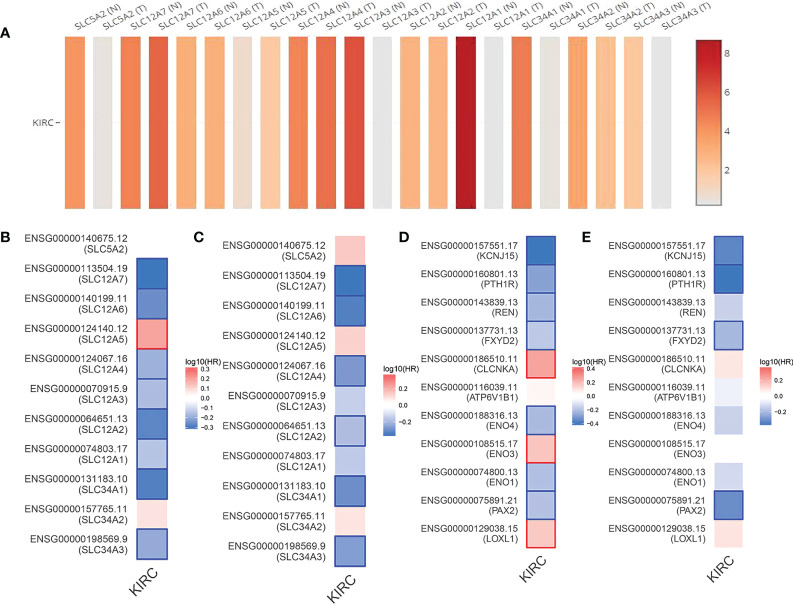
**(A)** Expression of 11 SLC members in ccRCC tissue samples and non cancerous tissue samples. **(B, C)** OS and DFS heat map analysis of 11 SLC members. **(D, E)** OS and DFS heat map analysis of the remaining coexpressed genes.

**Figure 6:**
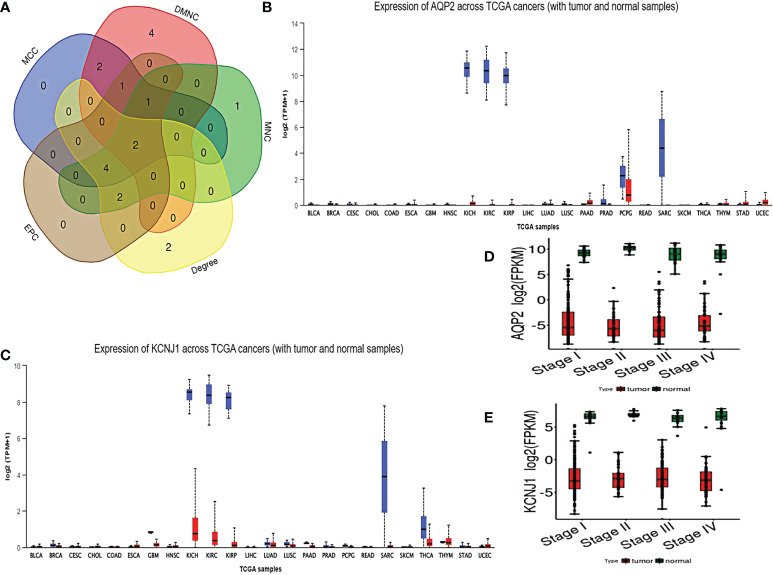
**(A)** using cytohubba plug-in to carry out five algorithms for hub genes, and select the top 10 genes for veen diagram analysis. **(B, C)** Pan cancer analysis of AQP2 and KCNJ1. **(D, E)** expression of AQP2 and KCNJ1 in various stages of ccRCC.

As shown by the co-expression analysis, the SLC family was closely related to the hub genes. Besides, SLC family members were further analyzed. According to the analysis on 11 SLC members, 6 SLC members were highly expressed in non-cancerous tissues (SLC5A2, SLC12A1, SLC12A3, SLC34A1, SLC34A2, SLC34A3), while SLC12A5 and SLC12A7 were highly expressed in ccRCC tissues (**Figure 5A**). As shown by survival heat map analysis, SLC12A1-A7, SLC34A1 and SLC34A3 were closely related to OS (**Figure 5B**), whereas SLC12A2/A4/A6/A7 and SLC34A1/A3 were closely related to DFS (**Figure 5C**). The heatmap of the remaining co-expressed genes showed a close relationship with the survival of ccRCC (**Figures 5D, E**). In order to further determine the more sensitive candidate genes, the cytohubba plug-in was adopted to obtain the top 10 hub genes through 5 algorithms, and then they were analyzed using venn map (**Table 2**). According to the results obtained, 2 overlapping genes are AQP2 and KCNJ1 (**Figure 6A**). Obviously, AQP2 was poorly expressed in KICH, KIRC, KIRP, PCPC and SARC (**Figure 6B**), while KCNJ1 was poorly expressed in KICH, KIRC, KIRP,SARC and THCA (**Figure 6C**). Fig C, D present the expression of AQP2 and KCNJ1 in each stage of ccRCC, with statistical differences in the results.

**Table 2:** DMNC, Degree, EPC, MCC, MNC top ten genes.

MCC	DMNC	MNC	Degree	EPC
AQP2	KCNJ1	KCNJ1	KCNJ1	KCNJ1
CLCNKB	C3	RHCG	ENO2	SLC12A1
KCNJ1	UMOD	SLC12A1	SLC12A1	SLC12A3
NPHS2	HRG	SLC12A3	SLC12A3	SLC34A1
SCNN1A	SCNN1B	SLC34A1	SLC34A1	UMOD
SLC12A1	SCNN1A	EGF	LOX	EGF
SLC12A3	AQP2	AQP2	EGF	AQP2
SLC34A1	PFKP	NPHS2	AQP2	NPHS2
SCNN1B	NPHS2	CLCNKB	CLCNKB	CLCNKB
UMOD	FBP1	PLG	PLG	PLG

## Discussion

RCC is the world’s 15th most common cancer whose incidence rate is obviously higher in the developed countries (9). The early clinical manifestations of RCC are unobvious, and metastasis tends to occur at the time of initial diagnosis. The huge economic burden of metastatic renal cell carcinoma is estimated at 1.6 billion US dollars in some countries, causing over 131,000 deaths and 342,000 deaths worldwide every year (10). According to the classification of the World Health Organization, RCC is classified into 16 subtypes. The most common subtype is clear cell ccRCC, accounting for about 75% (11). In the early stage of patients with metastatic RCC, the mortality rate increases due to the lack of effective diagnosis, and the resistance to treatment increases, triggering a huge economic burden. Therefore, it is urgent to find new biomarkers for early diagnosis and metastasis detection of ccRCC (12). In recent years, with the development of biological technology, researchers have explored the gene level changes of ccRCC, thus providing candidate gene sites for diagnosis and treatment.

In this study, five microarray data were analyzed to obtain DEGs in ccRCC and non-cancer tissue samples. Moreover, there were 118 differentially expressed genes, including 24 up-regulated genes and 94 down-regulated genes. Subsequently, the DEGs were enriched by GO and KEGG pathways. Indeed, GO analysis was mainly concentrated in excretion, ion homeostasis, cell protein metabolism, heparin binding, zinc binding, oxidoreductase activity, receptor binding, scavenger receptor activity, etc. Malignant tumors excreted many metabolites, including peptides in urine, which were altered by concentration in kidney. It could be used as a marker for the detection, prognosis and therapeutic target of ccRCC (13). Oxidoreductase, as one of the most important enzymes in the body, is involved in many biological processes, angiogenesis and cancer progression (14). According to other studies, the scavenger receptor is an important regulator of tumor behavior, which is associated with the immune response to cancer. At the same time, scavenger receptor can be used as a diagnostic biomarker and a new target for cancer interventions. It can be observed from the above results that the GO enrichment analysis of the DEGs is closely related to the development of tumors. Besides, KEGG pathway analysis is mainly concentrated in glycolysis/gluconeogenesis, thus collecting duct acid secretion, carbon metabolism, PPAR signaling pathway, etc. Peroxisome proliferator activated receptors (PPARs) are fatty acid activated transcription factors, which belong to nuclear receptor superfamily and regulate energy metabolism. According to some studies, it is closely related to the development of renal cell carcinoma (15). All these results suggest that the biological process and regulatory pathway of DEGs play an important role in tumor progression, and these genes can be used as candidate genes for diagnosis and treatment of ccRCC.

In order to further identify more sensitive candidate genes, 9 DEGs were screened as hub genes with degree greater than 10. Among the 9 hub genes, ENO2 and LOX were high expressed in ccRCC. ENO2 was closely related to OS, whereas LOX was closely related to DFS. In addition, ENO2 can affect the proliferation and apoptosis of renal cancer cells, which may not only reveal a new mechanism underlying development or progression of RCC but also identify new markers for its diagnosis and prognosis (16). Studies reported for the first time that the presence of LOX-1 protein in ccRCC urine and its peculiar distribution in tumoral tissues could lead to promising results in terms of diagnostic potential for ccRCC tumors (17). As shown by the survival curve of 9 hub genes, three members of the solute carrier family (SLC) are closely related to the OS of ccRCC. Currently, SLC superfamily includes 458 transport proteins from 65 families, which carry various substances across the cell membrane. Apart from that, SLC protein plays a role in the development and progression of cancer by regulating metabolic and environmental conditions (18). In this study, through the co-expression analysis of hub genes, a total of 11 SLC members were screened, namely SLC5A2, SLC12A1/A2/A3/A4/A5/A6/A7 and SLC34A1/A2/A3. As revealed by the survival heat map analysis, SLC12A1-A7, SLC34A1 and SLC34A3 were closely related to the OS of ccRCC, whereas SLC12A2/A4/A6/A7 and SLC34A1/A3 were closely related to DFS in ccRCC, which were significantly correlated with clinical stage, OS and DFS. Therefore, they may be the potential targets for clinical diagnosis, prognosis and treatment of patients with ccRCC. As pointed out by Kang, SLC22A6, SLC22A7, SLC22A13, SLC25A4, and SLC44A4 were closely associated with survival and prognosis of ccRCC (19).

In order to select the key genes, we used cytohubba plug-in to screen genes through five algorithms. Beyond that, 2 genes (AQP2, KCNJ1) were selected to analyze their relationship with tumor expression and clinical stage. Aquaporins (AQPs), as a family of water channels, also include a series of categories that mediate the transport of glycerol, ions and other molecules. Notably, AQPs can enhance cancer invasion and metastasis by promoting tumor angiogenesis, regulating protease and ECM degradation molecules, assisting to regulate epithelial mesenchymal transformation of cancer cells, and interacting with specific signal pathways (20). AQP2, as a classic aquaporin, is mainly expressed in renal cortex/medulla, gastric fundus, small intestine, pancreas islet and oviduct (21). According to the report of Moon CS, AQP2 is closely related to the invasiveness of endometrial cancer cells (22). Studies have revealed that AQP0/8/9/10 mRNA expression levels are up-regulated, whereas AQP1/2/3/4/5/6/7/11 mRNA expression levels are down-regulated in ccRCC. What’s more, high AQP0/8/9 expression was significantly associated with poor OS. On the contrary, high levels of AQP1/2/3/4/5/6/7/10/11, particularly high levels of AQP1/4/7, were associated with better OS in ccRCC (23). KCNJ1, as a potassium inward rectifier channel, plays a significant role in potassium homeostasis by transporting potassium out of cells. As claimed by Guo Z, KCNJ1, low-expressed in ccRCC and associated with poor prognosis, plays a crucial role in ccRCC cell growth and metastasis (24). Therefore, it is concluded from the above results that AQP2 and KCNJ1 can be used as target candidate genes for ccRCC.

In conclusion, this study is aimed to screen genes involved in the development of ccRCC. Firstly, a total of 118 DEGs and 9 hub genes can be used as markers for diagnosis and treatment of ccRCC. Secondly, through the co-expression analysis of hub gene, 11 SLC members may be related to the occurrence and development of ccRCC. According to the survival and expression analysis, 9 SLC members are closely associated with the survival rate of ccRCC. Finally, by screening the hub genes, this study found that 2 key genes are closely related to the progression of ccRCC. In short, these genes may provide a theoretical basis for the diagnosis and treatment of ccRCC. However, more evidence is needed to confirm the biological function and regulatory mechanism of these genes in ccRCC.

## Data availability statement

The datasets presented in this study can be found in online repositories. The names of the repository/repositories and accession number(s) can be found in the article/supplementary material.

## Author contributions

LP, QW and ZC participated in the design of the study and analysis of data. LP and LB designed the study and drafted the manuscript. JW, LF, DX and SY got involved in data collection. All authors contributed to the article and approved the submitted version.

## Funding

This study was supported by the Research Foundation of Anhui Medical University (No.2021xkj051, 2021xkj164), Clinical Scientific Research Cultivation Project of the Second Hospital of Anhui Medical University (No. 2020LCZD03, 2021LCZD04).

## Conflict of interest

The authors declare that the research was conducted in the absence of any commercial or financial relationships that could be construed as a potential conflict of interest.

## Publisher’s note

All claims expressed in this article are solely those of the authors and do not necessarily represent those of their affiliated organizations, or those of the publisher, the editors and the reviewers. Any product that may be evaluated in this article, or claim that may be made by its manufacturer, is not guaranteed or endorsed by the publisher.
